# Mild endoplasmic reticulum stress alleviates FB1-triggered intestinal pyroptosis via the Sec62-PERK pathway

**DOI:** 10.1007/s10565-024-09868-3

**Published:** 2024-05-21

**Authors:** Li Ma, Zhengqing Li, Dongmei Yue, Jie Qu, Ping Zhang, Shuxia Zhang, Kehe Huang, Yinuo Zou, Chunfeng Wang, Xingxiang Chen

**Affiliations:** 1 College of Veterinary Medicine, Nanjing Agricultural University, Nanjing, 210095 Jiangsu Province China; 2 Institute of Nutritional and Metabolic Disorders in Domestic Animals and Fowls, Nanjing Agricultural University, Nanjing, 210095 Jiangsu Province China; 3 MOE Joint International Research Laboratory of Animal Health and Food Safety, College of Veterinary Medicine, Nanjing Agricultural University, Nanjing, 210095 Jiangsu Province China; 4 College of Animal Medicine, Jilin Agricultural University, Changchun, 130118 Jilin Province China

**Keywords:** Fumonisin B1, Sec62-PERK pathway, Endoplasmic reticulum stress, Pyroptosis

## Abstract

Fumonisin B1 (FB1), a water-soluble mycotoxin released by Fusarium moniliforme Sheld, is widely present in corn and its derivative products, and seriously endangers human life and health. Recent studies have reported that FB1 can lead to pyroptosis, however, the mechanisms by which FB1-induced pyroptosis remain indistinct. In the present study, we aim to investigate the mechanisms of pyroptosis in intestinal porcine epithelial cells (IPEC-J2) and the relationship between FB1-induced endoplasmic reticulum stress (ERS) and pyroptosis. Our experimental results showed that the pyroptosis protein indicators in IPEC-J2 were significantly increased after exposure to FB1. The ERS markers, including glucose-regulated Protein 78 (GRP78), PKR-like ER kinase protein (PERK), and preprotein translocation factor (Sec62) were also significantly increased. Using small interfering RNA silencing of PERK or Sec62, the results demonstrated that upregulation of Sec62 activates the PERK pathway, and activation of the PERK signaling pathway is upstream of FB1-induced pyroptosis. After using the ERS inhibitor 4-PBA reduced the FB1-triggered intestinal injury by the Sec62-PERK pathway. In conclusion, we found that FB1 induced pyroptosis by upregulating Sec62 to activate the PERK pathway, and mild ERS alleviates FB1-triggered damage. It all boils down to one fact, the study provides a new perspective for further, and improving the toxicological mechanism of FB1.

**Graphical Abstract:**

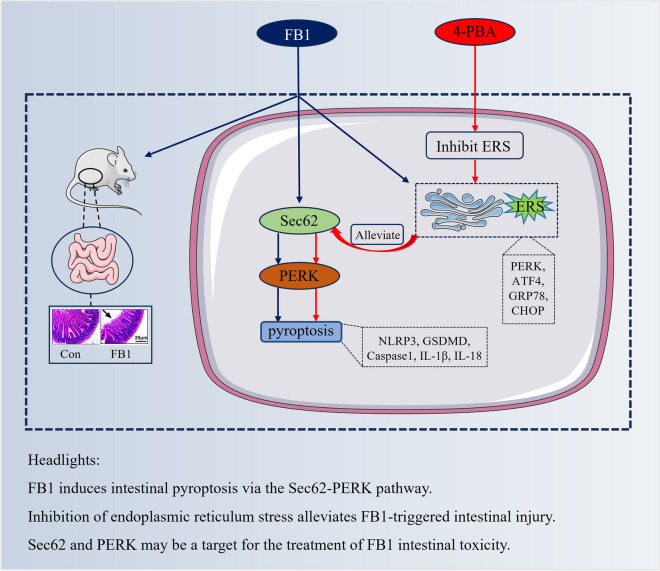

## Introduction

Fumonisins (FUMs) are water-soluble mycotoxins released by *Fusarium spp*, which commonly exist in corn (Dopavogui et al. 2023; Yang et al. 2023). However, fumonisin B1 (FB1) is the most toxic one of the fumonisins and causes serious harm to both human and animal health (Chen et al. 2023; Ezekiel et al. 2022; Yu et al. 2021). It triggers a diversity of damage in the body, including hepatointestinal injury, pulmonary edema, inflammatory response, and other forms of diseases (Chen et al. 2023; Gao et al. 2023). More recently, it was reported that FB1 induced cell apoptosis and caused organ damage by activating oxidative stress (Cao et al. 2022). Although researchers have gradually learned the toxicological mechanisms of FB1, the underlying mechanisms by which induced have not been fully elucidated.

Programmed cell death, pyroptosis, is a novel cell death form, that can be distinguished from other programmed cell death due to it depends on the gastrin protein, which is cleaved and activated by inflammatory caspases (Shi et al. 2017; Zhang et al. 2021). Considering a great deal of inflammatory factors were released in this process, pyroptosis also named inflammatory necrosis (Jorgensen et al. 2016). The classic pyroptotic pathway starts with caspase1, which can cleave gasdermin D (GSDMD) and further active GSDMD, eventually perforating the cell membrane and triggering cell rupture and necrosis (Chen et al. 2016). FB1, as a toxic stimulate factor, can trigger abnormal expression of TNF-α and inflammatory factors, including IL-1β (Interleukin-1 bata), IL-2 (Interleukin-2), IL-4 (Interleukin-4), and IL-10 (Interleukin-10), and cause intestinal inflammation in mice (Li et al. 2020). Likewise, FB1 can also disrupt intestinal porcine epithelial cells (IPEC-J2) tight junction, and induce barrier function impairment in IPEC-J2 (Chen et al. 2019; Goossens et al. 2012). FB1 causes significant inflammation and intestinal damage, however, whether it can lead to pyroptosis in the intestine and the underlying toxicological mechanisms need to be further explored.

Endoplasmic reticulum stress (ERS) is a compensatory behavior of cells, it happens when the imbalance of endoplasmic reticulum (ER) quality control occurs, including the proteins misfolde and unfolde that are accumulated, and the imbalance of Ca^2+^ in the ER lumen (Ma et al. 2023; Yue et al. 2023). At the same time, the unfolded protein response (UPR) is activated which will effectively decrease the production of misfolded and unfolded protein, then alleviates ERS UPR receptors located on the ER membrane, including dual kinase/endoribonuclease inositol-requiring enzyme 1α (IRE1α), kinase PKR-like ER-resident kinase (PERK), and activating transcription factor 6α (ATF6α), which can effectively monitor the degree of ERS and interact with its downstream molecules to play a crucial role in the process of ERS (Frakes and Dillin 2017; Oka et al. 2022). Preprotein translocation factor (Sec62), an ER-resident autophagy receptor, similarly, involved in the restoration of ER homeostasis (Fumagalli et al. 2016). Sec62 binds to LC3 and specifically transfers excess ER fragments to autophagosomes after ER stress, thereby maintaining a stable ER environment (Loi et al. 2019). Of note, FB1 exposure could activate ERS through the IRE1α axis and eventually trigger mitochondria-dependent apoptosis (Liu et al. 2020). Yu et al*.* demonstrated that the ERS-related PERK-CHOP signaling pathway was involved in FB1-induced gastrointestinal injury (Yu et al. 2020a, b). However, the mechanisms of FB1-induced injury to intestinal cells and organs and whether upstream that includes ERS participated is not yet clear.

Here, we investigate how FB1 triggers pyroptosis in mice intestine and IPEC-J2, and how ERS as an upstream regulates FB1-induced intestinal injury. Our results further implicate the mechanism toxic of FB1.

## Materials and methods

### Reagents and chemicals

Twelve male C57BL/6 mice were purchased from the Experimental Animal Center of Yangzhou University (Yangzhou, China), the age of the mice is between 42–56 days, and their weight is about 18–22 g. China Institute of Veterinary Drug Control (Nanjing, China) provided IPEC-J2 cells. Pribolab Bioengineering Co., Ltd. (Qingdao, China) synthesized FB1. Sigma Chemical Company (Missouri, USA) provided Thiazolyl blue (MTT) and Sodium 4-phenylbutyrate (4-PBA). Dingbei Chemical Company (Nanjing, China) provided the dimethyl sulfoxide (DMSO), DMEM-F12, and 4’,6-diamidino-2-phenylindole (DAPI). Gibco (Massachusetts, USA) provided FBS fetal bovine.. SYBR Premix Ex Taq kit and Sec62 antibody were obtained from Takara (Shanghai, China) and Abcam (Cambridge, UK), respectively. All of the antibodies including β-actin, GAPDH, NLRP3, GSDMD, Caspase1, IL-1β, IL-18, PERK, p-PERK, GRP78, and Cell Signaling Technology Company (Massachusetts, USA) provided LC3BI/II. ECL chemiluminescence detection kit was from purchased Biosharp (Hefei, China).

### Animals and experimental design

All of the animals were placed in the environment (lighting time: 12 h/day, temperature: 23 ± 1℃, humidity: 60 ± 10%). The adaptive phase was one week for animals to adapt to laboratory living conditions (standard chow from Jiangsu, China). Animals were assigned to the control group and FB1 group randomly, with 6 experimental mice in each group. Mice in the FB1 group were fed with FB1 dissolved 0.1 M in standard NaHCO_3_ solution every other day for half a month, while the dose of FB1 was 2.5 mg/kg/body weight, the dose and duration of FB1 exposure for mice were confirmed according to the preliminary experiment and the article from Mao et al. (Mao et al. 2023) and Cao et al. (Cao et al. 2020). Mice in the control group were treated with a standard NaHCO_3_ solution, intestinal tissue was collected for subsequent experiments. Animal experiments were performed based on the Guide for the Care and Use of Laboratory Animals published by the National Institutes of Health (Bethesda, MD, USA) and approved by the Laboratory Animal Care and Use Committee of Nanjing Agricultural University (certificate ID: SYXK (Su) 2011–0036).

### Histopathological analysis

After collecting the intestinal tissue samples, pathological tissue sections were made (H&E). For the details see Mao et al. (Mao et al. 2022).

### Cell culture

IPEC-J2 cells were cultivated in DMEM-F12 medium (10% FBS, 1% penicillin–streptomycin) and placed in the cell incubator (37 °C, 5% CO_2_). 3 × 10^5^ cells per well were cultured in plates for 24 h.

### The cytotoxicity assay by MTT

4 × 10^3^ cells per well were grown in 96-well plates which were exposed in FB1 (1–64 μM) for 24 h, 5 independent experiments, and negative controls were set for each concentration. After 24 h, 15 μL (5 mg/mL) of MTT solution was treated. After incubating at 37 °C for 4 h, add 200 μL DMSO to each well, and shake gently for 3 min. Finally, the GENios microplate reader tests OD value at 490 nm. Repeat the above steps to complete the cell viability test of 36 h and 48 h treatment. Based on these results, the cytotoxic dose of FB1 was identified and used in subsequent experiments.

### Quantitative real-time PCR (qRT-PCR)

After culture the cells in 12-well plates. Incubate with a new serum-free medium containing 8 μM FB1 for 24 h, 36 h, and 48 h, respectively. Samples processed at different time points were collected at the same time. After FB1 treatment, cells were washed twice with pre-cooled PBS. Then, the qRT-PCR detail steps were reported in our previous study (Mao et al. 2022). The primers used are shown in Table 1.

**Table 1:** Gene primers sequence

Target genes	Forward (5′-3′)	Reverse (5′-3′)
Porcine		
*NLRP3*	GACCTCAGCCAAGATGCAA	GACCTCAGCCAAGATGCAA
*GSDMD*	TTCATGGTTCTGGAGACCCC	TCATGGAAGTAGAAGGGGCC
*Caspase1*	CAGGAGTCCTCGAACTCTCCACAG	GGCTCTGAAGACGCAGGCTTAAC
*IL-1β*	TCTCTCACCCCTTCTCCTCA	TCTCTCACCCCTTCTCCTCA
*IL-18*	TCATGGAAGTAGAAGGGGCC	TCATCAATATTATCAGGAGGACTCATTT
*GRP78*	AAGGTCTATGAAGGTGAGCG	AGGCGATTTTGGTCATTGGTA
*PERK*	GCTATATATGAGCCCAGAGCAGAT	CATCTGAGTGCCGAATGGGTA
*ATF4*	AGACAACAGCAAGGAGGATG	ATTGGGTTCACTATCTGCGG
*CHOP*	CCTGGAAATGAGGAGGAGTC	CCTGGGTCTTCTTTGGTCTT
*Sec62*	CACAAGAAGCGGATTCAGGAAGT	ACCCGGTGACCCATCATATT
*β-actin*	TCACAAGACCAGGCATATTCTT	AAAGAAAGGGTGTAAAACGCA
Mouse		
*NLRP3*	TACTTGGGTGAAAATGCCCT	CAGAATTCACCAACCCCAGT
*GSDMD*	AACGATGTGTTCGTGGTGA	GGAAGAGAAGGATGTCCCAG
*Caspase1*	AGAGGATTTCTTAACGGATGCA	TCACAAGACCAGGCATATTCTT
*IL-1β*	GCCCTGTACCCCAACTGGTA	CCTCTGGGTATGGCTT
*IL-18*	GCTGAAAACGATGAAGACCTG	ATGGTTACTGCCAGACCTCTA
*GRP78*	GATAATCAACCAACTGTTAC	GTATCCTCTTCACCAGTTGG
*PERK*	GCTCAAAGACGAAAGCACAGAC	CCCACCGAGAAAGACCGAC
*ATF4*	GAGTAATGTAAGCAGCAGAGTCAGG	ATTCCTTTAGTTTAGAGCTAGGCAGTG
*CHOP*	GTTCTCCTGCTCCTTCTCCTTCAT	TACTCTTGACCCTGCGTCCCTAG
*Sec62*	CTCCTTGCTATTGCTCGCTG	TGACTTTTCCTCACTGTCGG
*β-actin*	CTAGGCACCAGGGTGTGATG	GTACATGGCTGGGGTGTTGA

### Western blotting (WB)

Cells were incubated for 24 h (at 2 × 10^5^ cells per well, 6-well plates). Subsequent steps are the same as the above-mentioned 12-well plate treatment of cells. For the following steps, see Mao et al.'s study (Mao et al. 2022).

### Immunofluorescence

Circular glass slides with a diameter of 20 mm were placed in 12-well plates, and 8 × 10^4^ cells were seeded per well. After treatment grouped according to the respective assay, washed 3 times with PBS, and incubated in specific anti-rabbit polyclonal antibody GRP78 (1/200 dilution) for 12 h, during this time the mixture was stored at 4 °C. Next, the round coverslip was used PBS to wash 3 times again, after the step, incubated in FITC-labeled antibody for 1 h in the incubator, and then cultured in DAPI for 5 min. Electron images were observed using a Laser Scanning Confocal microscope (Zeiss LSM 510; ZEISS Group; Germany).

### Cell transfection assay

Culture cells in a 12-well plate. After the cells reached 70% confluence, the cell transfection experiment was carried out. Plasmid Sec62-siRNA and PERK-siRNA were added to the DMEM-F12 medium and mixed gently, and then TransIntro TMEL reagent was added and standing for 20 min, the mixture was added to each well incubated for 6 h. Then, after changing the new medium and continuing to culture for 36 h, the cell samples were collected for subsequent experimental detection. The transfection experiment of plasmid GFP-LC3BII-DNA was completed with the above steps.

### The treatments with inhibitors

Before FB1 exposure, pretreate each well of the cells with 4-PBA at a concentration of 10 mmol/L for 4 h.

### Statistical analysis

One-way ANOVA followed by Duncan’s multiple was used to test the data. There are 3 independent experiments data are presented as mean ± SD (standard deviation). The statistical analysis and histograms were performed using SPSS and GraphPad Prism (The version of the software is 8.0) respectively. Statistically significant the results were considered at *P* < 0.05.

## Results

### FB1 activates the ERS-PERK pathway, induces pyroptosis, and causes intestinal villus damage in mouse intestinal tissue

**Fig. 1:**
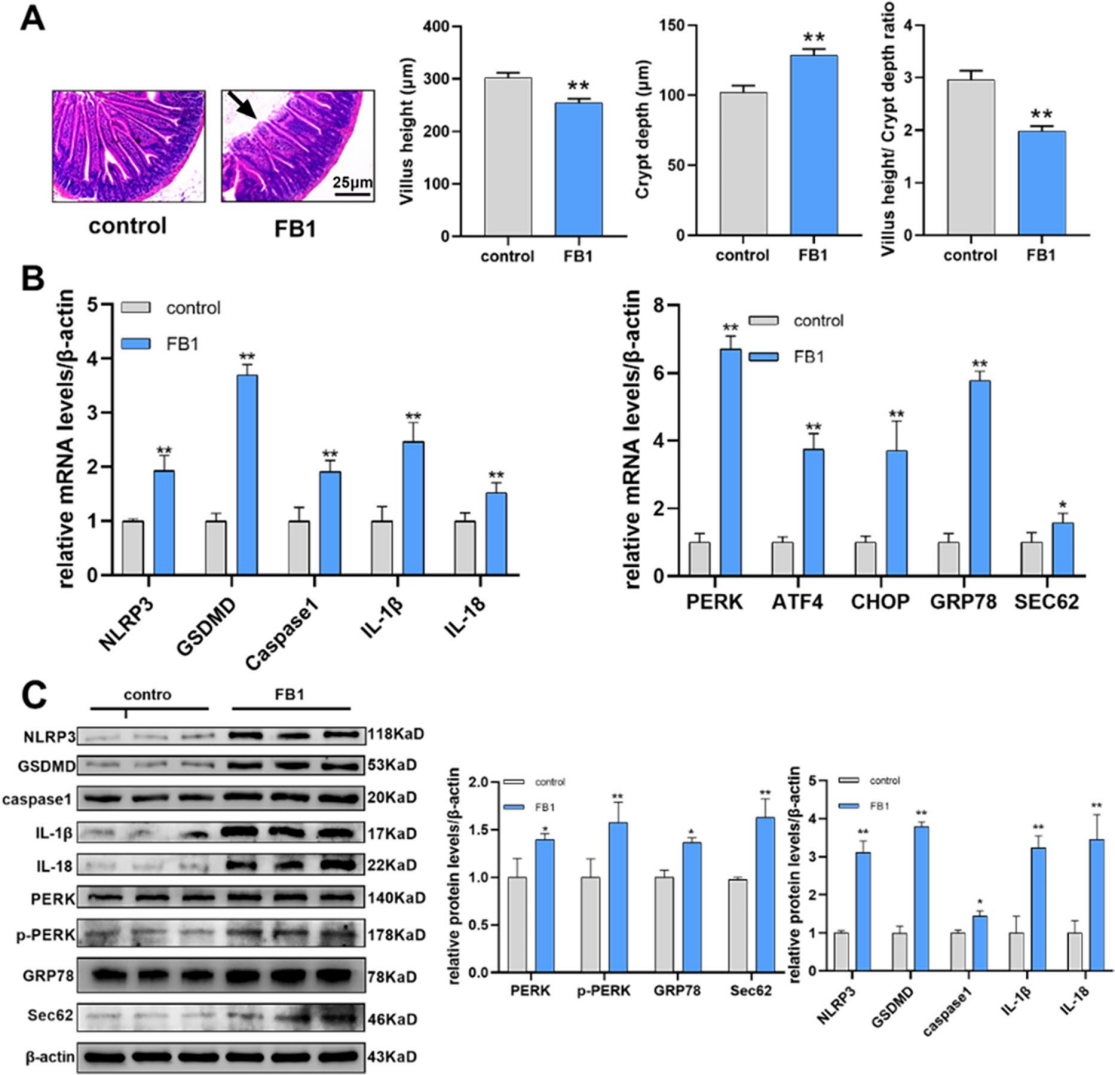
FB1 induces pyroptosis and ERS and causes intestinal villus damage in mouse intestinal tissues. (**A**) HE staining tests the morphology of the mouse intestinal villus and measures the length of the intestinal villus and the position of the crypt (*n* = 6). (**B**) qRT-PCR test mRNA levels of *NLRP3*, *GSDMD*, *Caspase1*, *IL-1β*, *IL-18*, *PERK*, *ATF4*, *CHOP*, *GRP78*, and *Sec62*. (**C**) Relative protein expression of NLRP3, GSDMD, Caspase1, IL-1β, IL-18, PERK, p-PERK, GRP78, and Sec62 were measured using WB. Data (*n* = 3) were presented as mean ± SD, * shows the significant differences at* p* < 0.05, and ** at *p* < 0.01, the same as following

The mice were sacrificed 2 weeks after FB1 administration and intestinal tissues were collected. HE staining was conducted to evaluate the severity of the gut injury, the results showed that after FB1 exposure, the intestinal villus of mice was swollen, the top of the villi was damaged and ruptured, the length of the intestinal villus was significantly shortened and the crypts were significantly moved down comparing with the control group (Fig. 1A). The mRNA (Fig. 1B) and protein (Fig. 1C) levels of Caspase1, and pyroptosis-related markers that are NLRP3 and GSDMD, and inflammatory markers that are IL-1β, and IL-18 were significantly higher in FB1 group than in control group. Therefore, severe pyroptosis and inflammation occurred in the intestinal tissues of the mice in the FB1 group. Similarly, the relative mRNA levels (Fig. 1B) of ERS markers, including *PERK*, *ATF4*, *CHOP*, *GRP78*, and ER-phagy receptor *Sec62* were also up-regulated in the FB1 group with statistical significance. Western blotting results showed that the protein expression (Fig. 1C) of PERK, p-PERK, GRP78, and Sec62 was also significantly increased, indicating that FB1 can induce ERS in the mouse intestine.

### FB1 induces pyroptosis in IPEC-J2 cells

**Fig. 2:**
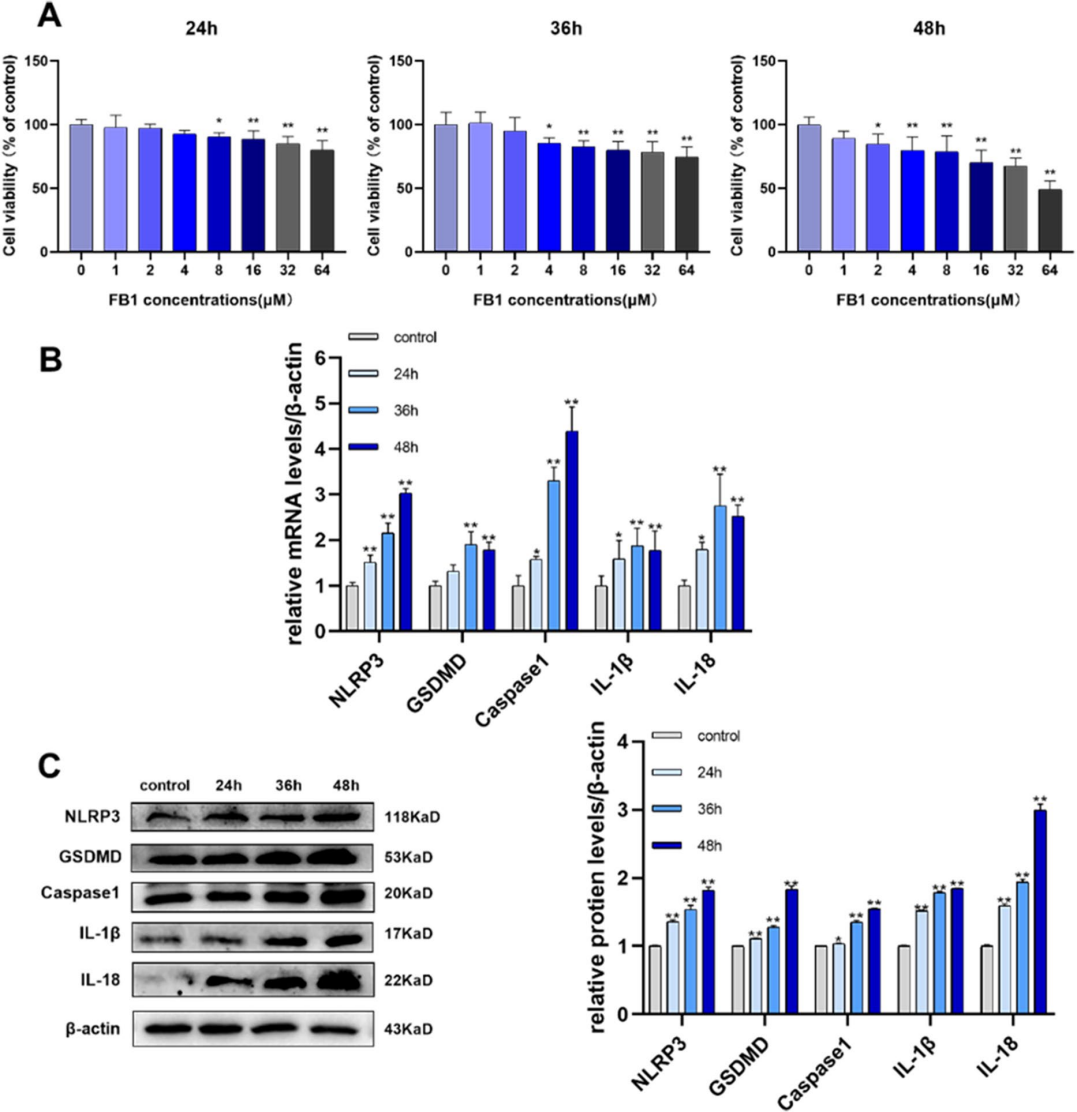
FB1 induces pyroptosis in IPEC-J2 cells. (**A**) after 24, 36, and 48 h of treatment with FB1 (8 μM), cell viability was measured (*n* = 6). (**B**-**C**) mRNA and protein levels of NLRP3, GSDMD, Caspase1, IL-1β, and IL-18 were measured by qRT-PCR and WB respectively

The mechanisms by which FB1-challenged in IPEC-J2 in *vitro* were further explored. We adopted the MTT assay to evaluate the toxic effects of FB1 on IPEC-J2 and observed the viability level of cells among different groups. We treated cells with different FB1 concentrations and at different times to determine the threshold concentration inducing a significant reduction in cell viability. According to the MTT results, (Fig. 2A). Cell viability was significantly decreased after treatment with FB1 8–64 μM for 24 h, 4–64 μM for 36 h, and 2–64 μM for 48 h. We treated cells with 8 μM FB1 for subsequent experiments (The time of the experiment is 24 h, 36 h, and 48 h). qRT-PCR results showed the markers of *NLRP3*, *GSDMD*, *Caspase1*, *IL-1β*, and *IL-18* relative mRNA expression levels (Fig. 2B) were significantly increased after exposure to FB1. When FB1 was exposed in a time-dependent manner, the protein levels (Fig. 2C) were significantly increased. Thus, we confirmed that FB1 induced pyroptosis in IPEC-J2 cells.

### FB1 activates the PERK pathway and upregulates Sec62 in IPEC-J2 cells

**Fig. 3:**
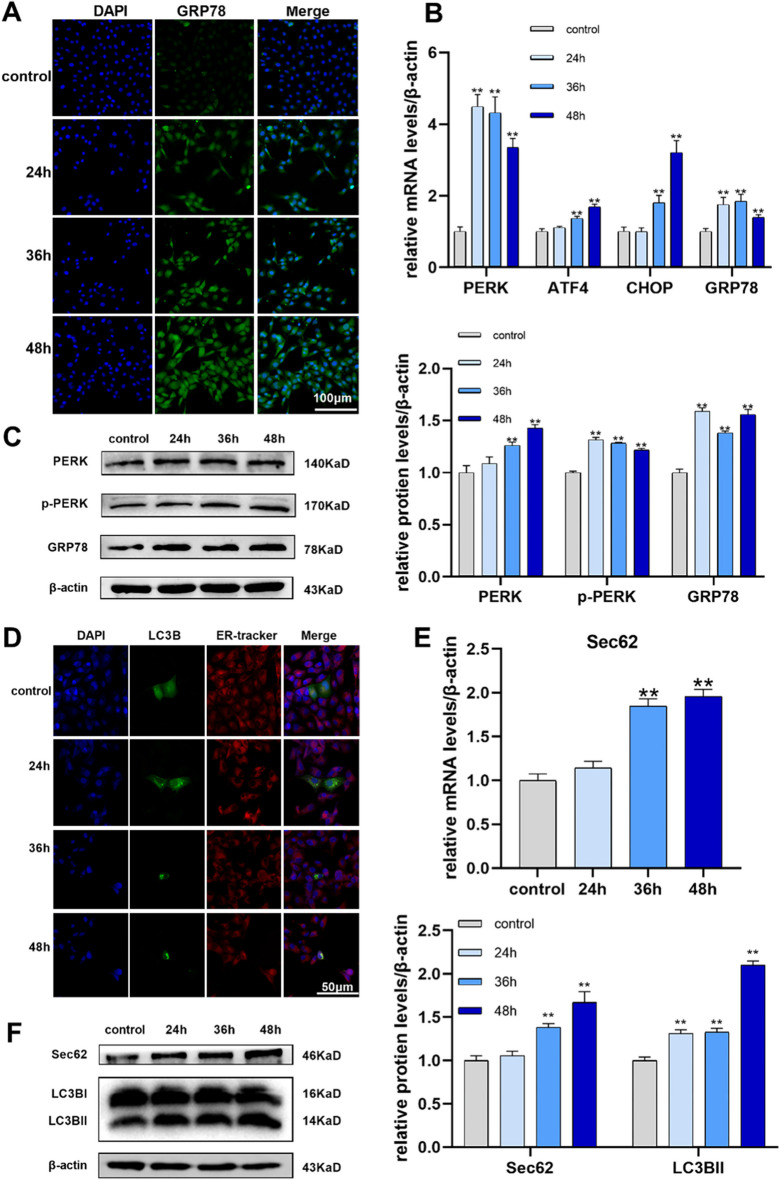
FB1 activates PERK pathway, and upregulates Sec62 in IPEC-J2 cells. (**A**) Immunofluorescence staining (The scale bar of the image is 100 μm and the original magnification is 400 ×) of GRP78 protein in IPEC-J2 cells, blue fluorescence indicates the nucleus, and green fluorescence indicates GRP78 protein. (**B**) mRNA levels of *PERK*, *ATF4*, *CHOP*, and *GRP78* were detected by qRT-PCR. (**C**) Protein relative expression levels of PERK, p-PERK, and GRP78 were measured by WB in cells transfected with LC3 plasmid. (**D**) Fluorescent regions of LC3B protein were visualized by LSCM (scale bar: 50 μm. Original magnification, 400 ×). The blue fluorescence is the nucleus, the green fluorescence is GFP-LC3B, and the red fluorescence is the ER tracker. (**E**) The mRNA level of *Sec62* was measured by qRT-PCR. (**F**) Protein levels of Sec62 and LC3BI/II were measured by WB. FB1 concentration: 8 μM

To determine whether FB1 induces ERS in IPEC-J2, the samples were collected after FB1 exposure for 24 h, 36 h, and 48 h, respectively. Immunofluorescence was adopted to evaluate the protein levels of GRP78, an ERS marker. Blue fluorescence (Fig. 3A) indicates the nucleus and green fluorescence indicates GRP78 protein expression. The green fluorescence intensity of FB1 was significantly enhanced than that in the control group. In addition, the mRNA levels of ERS markers (*PERK*, *ATF4*, *CHOP*, and *GRP78*) were also significantly up-regulated (Fig. 2B). Moreover, the protein levels of PERK, p-PERK, and GRP78 were higher than control group in a time-dependent manner (Fig. 3C). Sec62 is an ER-resident membrane protein (also known as ER phagocytosis receptor) and may have a regulatory effect on PERK. As an ER phagocytosis receptor, the elevated expression of Sec62 aggravates ER phagocytosis. We detected phagosomes with GFP-LC3B in the ER using immunofluorescence. The blue fluorescence is the nucleus, the green fluorescence is GFP-LC3B, the red fluorescence is the ER tracker, and the yellow fluorescence that appears in the merge is the phagosome formed by the ER phagocytosis receptor. The number of ER phagosomes was significantly increased after FB1 exposure compared with controls (Fig. 3D). The mRNA and protein levels of Sec62 were significantly increased after FB1 exposure (Fig. 3E-F). Taken together, the PERK pathway and high expression levels of Sec62 mediate FB1 and induce ERS.

### FB1 activates the PERK pathway by upregulating the expression of Sec62

**Fig. 4:**
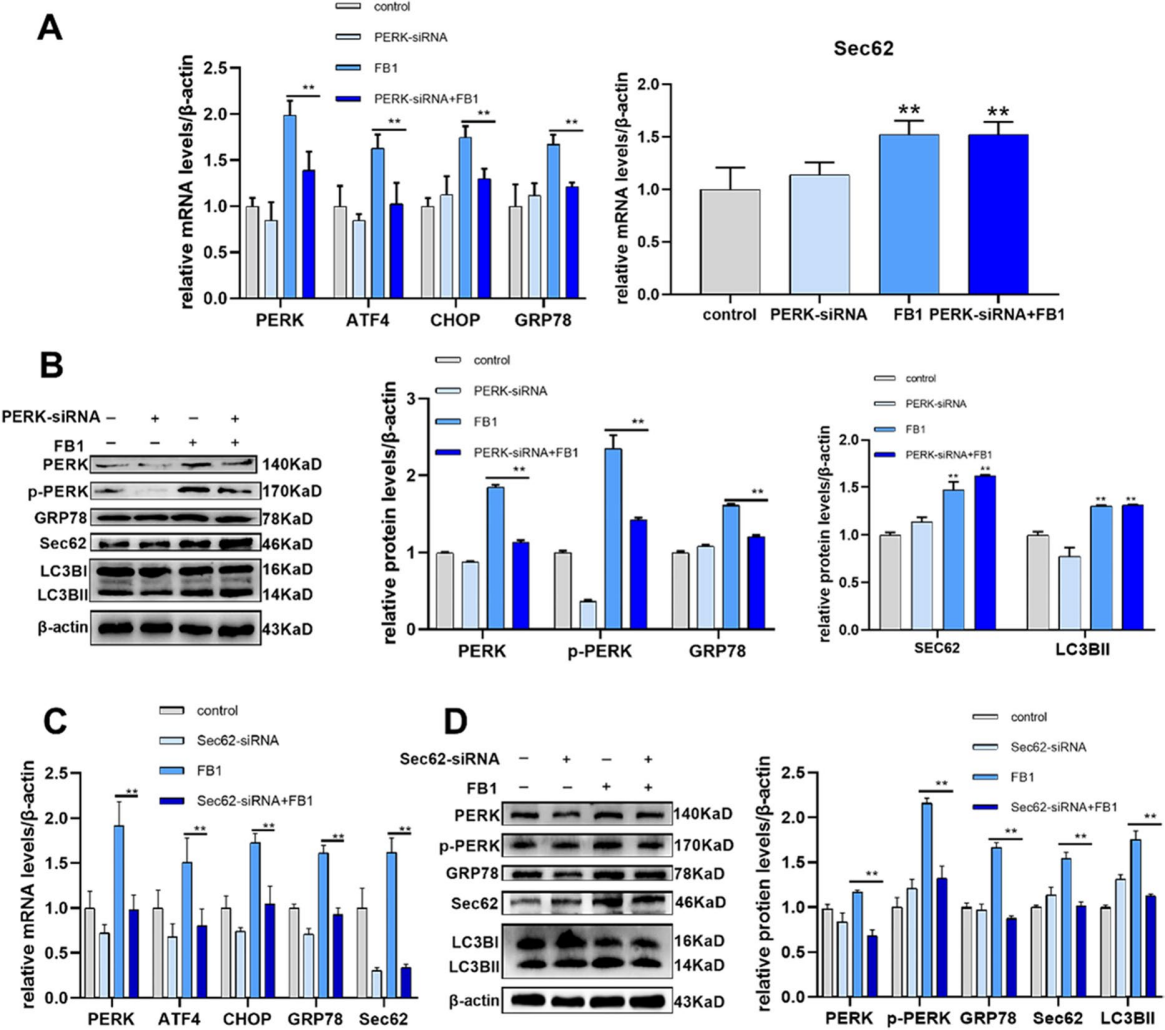
FB1 activates the PERK pathway by upregulating the expression of Sec62 in IPEC-J2 cells. (**A**) mRNA levels of *PERK*, *ATF4*, *CHOP*, *GRP78*, and *Sec62* were detected by qRT-PCR after transfecting PERK-siRNA. (**B**) Protein relative expression levels of PERK, p-PERK, GRP78, Sec62, and LC3BII were measured by WB after adding PERK-siRNA. (**C**) mRNA levels of *PERK*, *ATF4*, *CHOP*, *GRP78*, and *Sec62* were detected by qRT-PCR. (**D**) Protein levels of PERK, p-PERK, GRP78, Sec62, and LC3BII were measured by WB. FB1 concentration: 8 μM. PERK-siRNA (66.67 pmol/mL) and Sec62-siRNA (66.67 pmol/mL) were transfected for 6 h respectively

To further determine the regulatory relationship between Sec62 and PERK, we transfected cells with PERK-siRNA and Sec62-siRNA. After silencing the PERK with siRNA, the mRNA expression levels of *PERK*, *ATF4*, *CHOP*, and *GRP78* were significantly down-regulated compared with FB1 exposure only (Fig. 4A). The protein levels of PERK, p-PERK, and GRP78 were significantly down-regulated compared with the FB1-treated group (Fig. 4B). However, there was no significant difference in the mRNA and protein levels of Sec62 and LC3BII after transfecting PERK-siRNA (Fig. 4A-B). Likewise, after silencing Sec62 with Sec62-siRNA, the mRNA levels of *PERK*, *ATF4*, *CHOP*, and *GRP78* were significantly down-regulated compared with the FB1 group (Fig. 4C). The protein levels of PERK, p-PERK, GRP78, Sec62, and LC3BII were significantly down-regulated compared with the FB1 group (Fig. 4D).

### FB1 induces pyroptosis via Sec62-PERK pathway

**Fig. 5:**
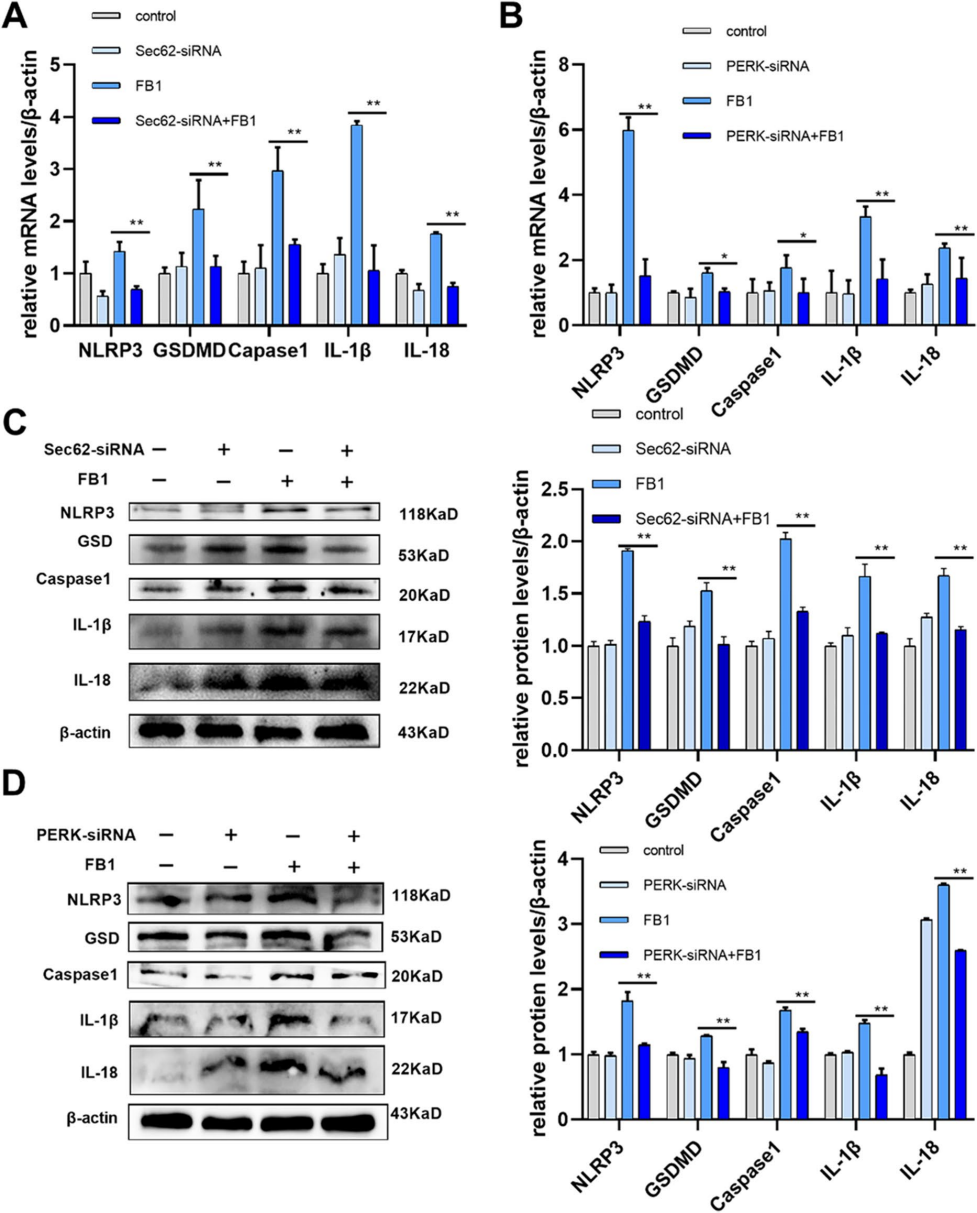
FB1 induces pyroptosis by Sec62-PERK pathway in IPEC-J2 cells. After silencing PERK and Sec62 with siRNA, the expression of mRNA (**A**, **B**) and protein (**C**, **D**) The methods of qRT-PCR and WB tested the levels of NLRP3, GSDMD, Caspase1, IL-1β, and IL-18. FB1 concentration: 8 μM. PERK-siRNA (66.67 pmol/mL) and Sec62-siRNA (66.67 pmol/mL) were transfected for 6 h respectively

To clarify the relationship between FB1-induced pyroptosis and the Sec62-PERK pathway. We compared the expression of pyroptosis-related markers in the silencing Sec62 group and the FB1 group. According to qRT-PCR and WB results, in the PERK-siRNA + FB1 group, the mRNA and protein relative expression levels of pyroptosis-related marks NLRP3, GSDMD, and Caspase1, and inflammatory-related factors IL-1β, and IL-18 were significantly lower than in FB1 group (Fig. 5A, C). Similarly, by silencing PERK, in the PERK-siRNA + FB1 group, the mRNA and protein expression levels that tested the same as described above were significantly lower than in the FB1 group (Fig. 5B, D). Taken together, the upregulation of either PERK or Sec62 is both the upstream of FB1-induced pyroptosis in IPEC-J2 cells.

### Inhibition of ERS can effectively alleviate the pyroptosis caused by FB1 via the Sec62-PERK pathway

**Fig. 6:**
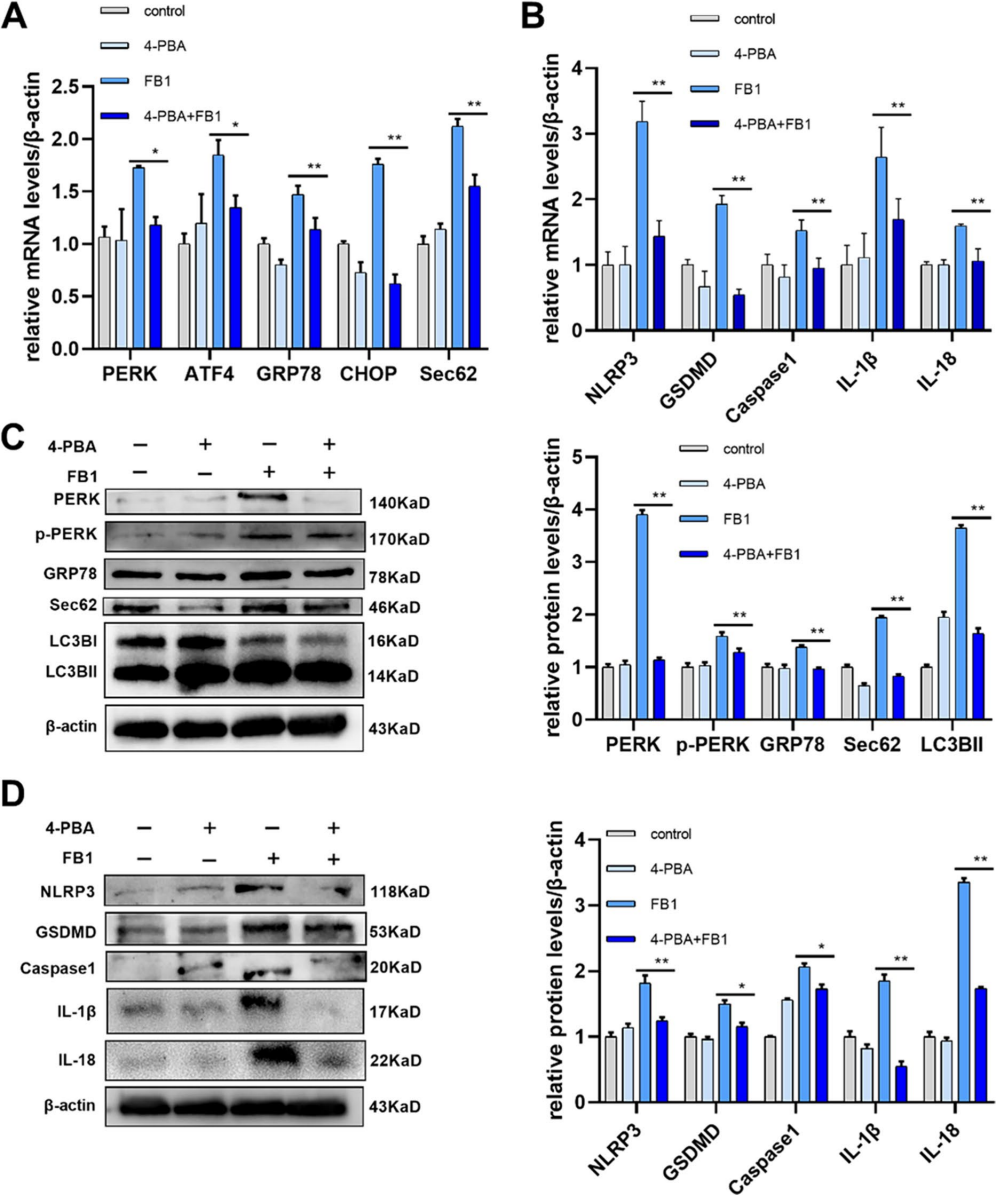
Inhibition of ERS can effectively alleviate the pyroptosis caused by FB1 via the Sec62-PERK pathway in IPEC-J2 cells. (**A**) The method of qRT-PCR tested the mRNA levels of *PERK*, *ATF4*, *GRP78*, *CHOP*, and *Sec62*. (**B**) The method of qRT-PCR tested the mRNA levels of *NLRP3*, *GSDMD*, *Caspase1*, *IL-1β*, and *IL-18*. (**C**) The method of WB tested the protein levels of PERK, p-PERK, GRP78, Sec62, and LC3BI/II. (**D**) Protein levels of NLRP3, GSDMD, Caspase1, IL-1β, and IL-18 were measured by WB. FB1 concentration: 8 μM. 4-PBA (10 mmol/L) was pretreated for 4 h

Numerous studies have confirmed that the ERS inhibitor 4-PBA can effectively inhibit ERS. Therefore, we pretreated cells with 4-PBA to observe whether it could alleviate the damage of FB1 on IPEC-J2 cells. The mRNA levels of *PERK*, *ATF4*, *GRP78*, *CHOP*, and *Sec62* were significantly down-regulated by qRT-PCR compared with FB1 alone (Fig. 6A) The protein levels of PERK, p- PERK, GRP78, Sec62, and LC3BI/II were also significantly down-regulated compared with the FB1 group (Fig. 6C). Pyroptosis indicators were significantly decreased compared with those treated in FB1 group. It was shown that in the 4-PBA + FB1 group, the mRNA and protein levels of Caspase1, and pyroptosis-related markers NLRP3, GSDMD, and inflammatory-related markers IL-1β, and IL-18 were significantly down-regulated compared with those in the FB1 group (Fig. 6B, D). In conclusion, inhibition of ERS can effectively decrease FB1-induced upregulation of Sec62, inhibit the PERK pathway, and alleviate pyroptosis.

## Discussion

FB1 brings harmful effects to animals, in the present study, our work investigated it-triggered enterocyte pyroptosis through the Sec62-PERK pathway in vitro and in vivo.

We found that FB1 induces ERS and pyroptosis in the intestine. Previous studies have already demonstrated that FB1 can induce intestinal pyroptosis by mTOR-mediated autophagy pathway, it can also lead to cell apoptosis, while few reports linked the ERS and pyroptosis to intestinal injury (Mao et al. 2022; Wang et al. 2021). Mice are the most commonly used animal models of intestinal diseases. Hence, we examined the pyroptosis markers, pyroptosis-related markers NLRP3, GSDMD, and Caspase1, and inflammatory-related markers IL-1β, and IL-18, the ERS markers, PERK, ATF4, CHOP, GRP78, and Sec62, and histopathological sections, uncovering the toxic effects of FB1. However, the deeper mechanisms need to be further explored.

In our study, we revealed PERK pathway is involved in regulating FB1-triggered pyroptosis. PERK, an RNA-dependent protein kinase (PKR)-like ER kinase, is a key ERS sensor in the UPR (Cubillos-Ruiz et al. 2017). It plays a critical role in ROS-mediated ERS, and the PERK-eIF2α-ATF4 axis directly leads to apoptosis (Liu and Zhang 2020; Verfaillie et al. 2012). FB1 cytotoxicity, as also mentioned above, was demonstrated to induce gastrointestinal injury by ERS-related PERK-CHOP signaling pathway (Yu et al. 2020a, b). Indeed, we used small interfering RNA silence of the PERK gene and found pyroptosis-related markers were downregulated, thus confirming that FB1 exposure promotes pyroptosis by activating the PERK pathway.

Our work further proved that Sec62 acts as an upstream regulator to PERK. As one of the components of the protein translocation machinery, Sec62 is an important ER transmembrane protein and functions in ER-phagy and facilitates protein transporting to the ER, while ER-phagy induced by Sec62 is one of the means to alleviate UPR (Linxweiler et al. 2017; Wu et al. 2021). Studies have shown that Sec62 can lead to autophagy by upregulating PERK/ATF4 expression and binding to LC3II (Su et al. 2022). In our work, we found that FB1 upregulated the expression of Sec62 and LC3BII. Through the bidirectional interference assay analysis of Sec62-siRNA and PERK-siRNA, we found that silencing the PERK did not affect the expression of Sec62 and LC3BII while silencing the sec62 gene could significantly inhibit the PERK pathway and expression of LC3BII. Notably, silencing either Sec62 or PERK can reduce the levels of ERS and pyroptosis-related markers.

Moreover, we demonstrated that inhibition of ERS alleviates the toxic damage induced by FB1 via the Sec62-PERK pathway. ERS is a protective physiological response of cells, and mild ERS can promote the recovery of cell homeostasis (Ma et al. 2023). Indeed, our study demonstrated that after inhibition of ERS with 4-PBA, did reduced PERK, pyroptosis, and inflammatory factor levels. Furthermore, several studies have shown that Sec62 can promote ER autophagy and reduce ERS (Wu et al. 2021). Conversely, the results of our study showed that the Sec62 level was decreased after ERS inhibition, indicating that ERS can regulate Sec62 expression. On these bases, we presume that there may be an ERS pathway acting as an upstream, which can regulate FB1-induced pyroptosis by the Sec62-PREK pathway, while the specific molecular mechanisms need to be further studied.

In our work, there are contains several limitations. First, we initially found that FB1 induces mice intestine pyroptosis, on the basis of this experiment, we further used cell experimentation including the counterproof methods to test the underlying mechanism behind it. However, because it has no Sec62 and PERK gene knockout mice were sold, and our team lack of the skill to knockout the gene in animals. Regrettably, it is hard for us to further confirm the findings in animal, if there are any data can be obtained to prove our findings, it would be better. Second, our study aims to investigate FB1-induced intestine toxicity, and until now, there is no any study shown that sex differences affect FB1-induced toxicity effects in the intestine. This is indeed a carelessness in our experiment design. But we don’t think it will affect the results of the animal and cell experiments.

## Conclusion

In summary, we demonstrated that FB1 through upregulating the expression of Sec62 and then activating the subsequent PERK contributes to pyroptosis in intestinal cells, and inhibition of ERS could reduce the damage. Our work further explores the molecular mechanisms of FB1 toxicity.

## Data Availability

Data will be made available on request.

## Abbreviations

*FB1*: (Fumonisin B1)
*ERS*: (Endoplasmic reticulum stress)
*ER*: (Endoplasmic reticulum)
*GRP78*: (Glucose-Regulated Protein 78)
*PERK*: (PKR-like endoplasmic reticulum kinase)
*ATF4*: (Transcription factor 4)
*CHOP*: (The C/EBP homologous protein)
*Sec62*: (Sec62 homolog, preprotein translocation factor)
*LC3BI/II*: (Microtubule-associated protein 1 light chain 3 beta)
*NLRP3*: (NOD-, LRR- and pyrin domain-containing 3)
*GSDMD*: (Gasdermin D)
*Caspase1*: (Cysteinyl aspartate specific proteinase 1)
*IL-1β*: (Interleukin-1β)
*IL-18*: (Interleukin-18)
*GAPDH*: (Glyceraldehyde-3-phosphate dehydrogenase)
*MTT*: (3-(4,5-Dimethyl-2-thiazolyl)-2,5-diphenyl-2-H-tetrazolium bromide)
*DAPI*: (4′,6-Diamidino-2-phenylindole)
*PVDF*: (Polyvinylidene fluoride)
*TBS*: (Tris-buffered saline)
*4-PBA*: (Sodium 4-phenylbutyrate)
*siRNA*: (Small interfering RNA)
*IPEC-J2*: (Intestinal porcine epithelial cells)
